# Use of a Web-based clinical decision support system to improve abdominal aortic aneurysm screening in a primary care practice

**DOI:** 10.1111/j.1365-2753.2011.01661.x

**Published:** 2012-06

**Authors:** Rajeev Chaudhry, Sidna M Tulledge-Scheitel, Doug A Parks, Kurt B Angstman, Lindsay K Decker, Robert J Stroebel

**Affiliations:** 1 Assistant Professor of Medicine; 2 Assistant Professor of Medicine; 3 Administrator, Employee and Community Health; 4 Assistant Professor of Family Medicine; 5 Lead Analyst Programmer, Clinical Systems; 6 Assistant Professor of Medicine, Division of Primary Care Internal Medicine, Center for Innovation, Department of Family Medicine and Information Technology, Mayo ClinicRochester, MN USA

**Keywords:** abdominal, aortic aneurysm, clinical decision support systems, delivery of health care, health care technology, patient-centred care, preventive health services

## Abstract

**Rationale, aims and objectives:**

The United States Preventive Services Task Force recommends a one-time screening for abdominal aortic aneurysm (AAA) with ultrasonography for men aged 65 to 75 years who have ever smoked. However, despite a mortality rate of up to 80% for ruptured AAAs, providers order the screening for a minority of patients. We sought to determine the effect of a Web-based point-of-care clinical decision support system on AAA screening rates in a primary care practice.

**Methods:**

We conducted a retrospective review of medical records of male patients aged 65 to 75 years who were seen at any of our practice sites in 2007 and 2008, before and after implementation of the clinical decision support system.

**Results:**

Overall screening rates were 31.36% in 2007 and 44.09% in 2008 (*P*-value: <0.001). Of patients who had not had AAA screening prior to the visit, 3.22% completed the screening after the visit in 2007, compared with 18.24% in 2008 when the clinical support system was implemented, 5.36 times improvement (*P*-value: <0.001).

**Conclusions:**

A Web-based clinical decision support for primary care physicians significantly improved delivery of AAA screening of eligible patients. Carefully developed clinical decision support systems can optimize care delivery, ensuring that important preventive services are delivered to eligible patients.

## Introduction

The prevalence of abdominal aortic aneurysm (AAA), a ballooning of a segment of a weak aortic arterial wall in the abdomen, is estimated to be 4% to 9% in men and 1% in women [1–7]. The prevalence of an AAA greater than 5.0 cm in men aged 50 to 79 years is estimated to be 0.5% [8]. An aneurysm that ruptures, leaking blood into the body, is fatal in 80% of cases [9]. AAA is the 14th leading cause of death in the USA, with as many as 9000 deaths occuring annually as a result of rupture of a 5-cm aneurysm [10, 11].

Ultrasound screening for AAA is an excellent screening test, with both a sensitivity and a specificity of over 95% [12]. The United States Preventive Services Task Force recommends a one-time screening for men aged 65 to 75 years who have ever smoked [13, 14].

Although there are standardized recommendations for all adult preventive services, the overall delivery of these services has not been optimal in the USA; up to 50% of patients do not receive age-specific, sex-specific preventive services or care for their chronic conditions [15,16].

The United States Preventive Services Task Force also recommends that primary care providers address preventive services during every patient visit, regardless of the reason for the visit [13]. However, during a typical 15-minute primary care visit, time constraints are a major limiting factor in physicians' ability to deliver preventive services. Experts estimate that physicians would have to work 18-hour days to address all recommended preventive and chronic care services for their panel of patients [17, 18]. As a result, only about half of these services are actually delivered [19, 20].

Clinical decision support technology saves physicians time and increases the likelihood that patients will get the care they need. The US government is currently promoting investments in health information technology (HIT), because it is estimated that only 17% of US physicians use even a basic electronic medical record (EMR) [21]. This figure is in sharp contrast to that of Europe, New Zealand and Australia, which boast physician EMR adoption rates of at least 80% [21]. Computerized clinical decision support, which reminds providers to give certain tests and treatments, can help in the delivery of recommended preventive services [22]. AAA screening is one example of such a preventative health service. In this study, we retrospectively examine the impact of a Web-based clinical decision support system in improving the AAA screening rate for eligible patients seen in our primary care clinics.

## Methods

### Practice setting

Mayo Clinic Rochester is a large, multi-specialty group practice in Rochester, MN. The primary care practices providing care to adult patients include internal medicine (primary care internal medicine) and family medicine. Forty-five general internists, 40 family physicians, 96 internal medicine residents and 20 family medicine residents provide care to nearly 115 000 adult patients at four sites in the Rochester area. For our primary care practices, we implemented a clinical decision support system in January 2008.

### Clinical decision support system – Generic Disease Management System

The clinical information systems at Mayo Clinic include a demographics registration system, a General Electric Centricity EMR, a gastrointestinal database and an allergies module. At the time of this study, the clinical decision support system for preventive services and disease management was not developed in our EMR. To address this need, VitalHealth Software, a joint venture between Mayo Clinic and the Netherlands-based Noaber Foundation, developed the Generic Disease Management System (GDMS) software.

The GDMS is a Web-based application that uses General Electric Web Services and a MSQweb.net platform to retrieve patient vital statistics such as blood pressure, weight, body mass index, age, demographic information, prior diagnoses, allergies, prior radiology diagnostic tests and previous preventive services (e.g. immunizations, cancer and metabolic screenings, laboratory test results pertaining to diabetes, coronary artery disease, asthma and depression) from different clinical information systems.

The GDMS includes a rules-based application coded with guidelines for age-specific, sex-specific preventive services and for process and outcome measures for diabetes and coronary artery disease. On the basis of the data from Web services, the rules provide point-of-care decision support regarding the services that the patient needs at their visit and in the next 90 days.

### Generic Disease Management System implementation and use for the primary care practices

**Figure 1:**
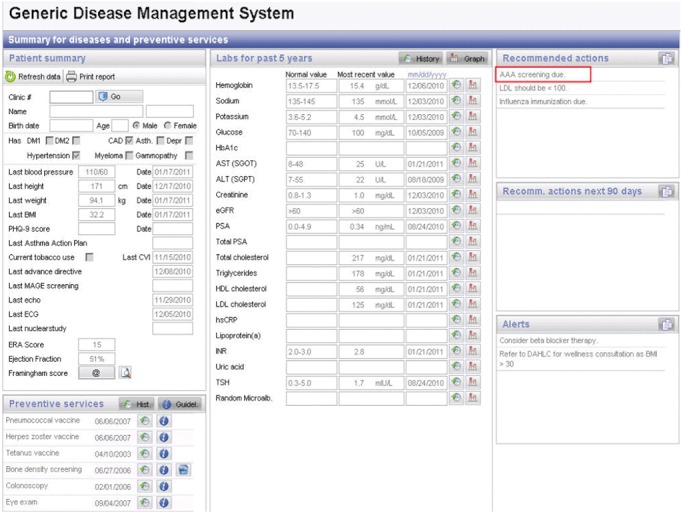
At patient visits, a paper copy of the Web-based system (Generic Disease Management System) summary screen is printed by check-in staff at the front desk. It is then included in the rooming packet for the allied health staff and providers to inform them of any necessary preventive services or tests.

The GDMS was developed and successfully pilot tested in December 2007, and the system was made available to all practice sites of the employee and community health practice for adults in January 2008. To fulfil practice needs to address all preventive services, the new workflow is that, when a patient visits a practice site for any reason (e.g. acute condition, chronic disease, annual examination), the desk staff (check-in staff) enter the patient's clinic number in the Web-based GDMS and print a paper copy of the GDMS summary screen (Fig. 1) at the front desk and it is included in the rooming packet for the allied health staff and providers. The provider can then address any preventive service or test for chronic conditions. In the case of AAA screening, they electronically order an ultrasound test for AAA in our orders system; at the end of the visit, the patient stops at the check-out desk, where the test is scheduled with the radiology department.

## Results

An independent data abstractor reviewed all the records of male patients aged 65 to 75 years who were seen at any employee and community health practice site in 2007 and 2008 and had ever used tobacco. The abstractor identified 883 such patients from 1 January to 31 March 2007 and 880 patients for the same period in 2008. In 2007, 29.55% of eligible patients had had a prior screening; in 2008, 31.82% had had a prior screening. The abstractor then checked which of the eligible patients had had an ultrasound of the aorta in the 30 days after their visit. In 2007, 20 (3.2%) patients had been screened for AAA; in 2008, 112 (18.3%) patients had AAA screening (*P* < 0.001; Table 1). Of the patients screened in 2008, three had an AAA larger than 5 cm, and one had an aneurysm of 3.5 to 4.5 cm. Two of the three patients whose aneurysm was larger than 5 cm underwent surgical repair.

**Table 1:** Patients receiving abdominal aortic aneurysm screening before and after clinical decision support system implementation

	2007 (before clinical decision support tool implementation)	2008 (after clinical decision support tool implementation)

Total number of patients (men aged 65 to 75 years who had ever smoked)	883	880
Number of patients who had screening before the visit	261	276
Number of patients eligible for the screening	622	614
Number of patients who received screening during the visit	20	112
Percentage of eligible patients screened (%)	3.2	18.3

## Discussion

In our study, the AAA screening rate among all eligible patients significantly improved after initiation of a Web-based point-of-care clinical decision support system (GDMS) in our primary care practice. The system's utilization in face-to-face encounters led to increased completion of screening after a clinic visit by identifying eligible patients and alerting their providers, who then engaged patients in the discussion, regardless of the reason for the visit. This was evidenced by a fivefold improvement in screening rates for eligible patients in 2008, when compared to the same period in 2007. This makes sense given that there are many tests that a patient might have for unrelated reasons that can also visualize the abdominal aorta; if the patient already had any of these tests, they do not need an ultrasound test for AAA screening. These tests include abdominal ultrasound, abdominal or pelvic computed tomography, magnetic resonance angiography of the abdominal aorta and computed tomography colonography.

Taking time out of a 15-minute primary care consultation to check the patient's EMR for a record of such a test is not possible. This is particularly true when the physician is seeing the patient for an acute condition (e.g. back pain, headache) and has a limited time to address it. The GDMS and our pre-visit work processes are designed to cue the provider if the patient has already had a test that visualized the abdominal aorta, saving us the time of searching for that information. Even though our study was active for only 3 months, five patients with AAA were identified, two of whom benefited from elective repair that may have saved their lives.

However, not all patients identified by the decision support as eligible for screening received it. In fact, 81.7% of eligible patients did not receive the screening. Although the system helped providers by identifying patients who needed preventive services and tests for chronic conditions, the limited time that providers have for visits might have been insufficient to address all the patients' needs. This highlights the importance of patient health records for enabling patients to be proactive in seeking the care they need. If our systems allowed patients to access their own version of GDMS before the visit (i.e. Web-based patient portal), they might have been proactive in asking for this service.

We have not investigated the possible reasons for lack of screening of eligible patients; however, we hypothesize that, because the GDMS alerts occur during all patient visits concerning any number of recommended tests or treatments unrelated to AAA, the providers might not have had time to address all the recommendations and therefore left them for a future visit. Recent literature also suggests that provider reminders have to be appropriately inserted into their workflow to have the desired effect [17]. A recent study showed that providers might ignore over 90% of alerts because of alert fatigue [18].

This finding underscores the importance of having standardized processes in place to support the clinical decision support system for services that are due. Primary care physicians have many tasks to perform during a 15- to 20-minute visit, and having other members of the health care team take responsibility for helping the physicians deliver the necessary services can lead to better results by getting the orders for the services ‘teed up’ for them [8].

With the upcoming changes spurred by health care reform and an increasing focus on quality and value, the emphasis on delivery of evidence-based preventive services and management of chronic conditions will place increasing demands on primary care providers. The decision support systems that help improve quality will play an increasingly important role. Without these systems, providers will not be able to provide high-value care to their patients, as they will need to determine the need for screening through a time-consuming and unreliable manual process.

As US efforts to improve health care quality and cap costs shift to HIT, carefully designed clinical decision support systems can optimize care delivery in primary care. As we move towards achieving cost-effective care and reducing unnecessary laboratory tests and radiologic imaging studies, it is important to note that our clinical decision support model enabled us to identify up to 31.92% of patients who had prior imaging of their aorta from an unrelated examination. This information helped us eliminate the need for an additional ultrasound examination, saving time and expense for patients and unnecessary cost for the organization and third-party payers.

As with any practice-based quality study, our study of the effect of decision support technology implementation had its limitations: (1) Our control population was retrospective from the prior year. We would have preferred a randomized controlled trial, but that was not feasible, because our goal was to provide improved care to all of our patients for all of their preventive services and chronic conditions. (2) We did not ask the primary care providers why the remaining eligible patients did not get the AAA screening as they were identified as needing. (3) We did not explore the relationship between the visit type and the likelihood of the patient getting the necessary screening.

## Conclusion

In conclusion, we observed an improved AAA screening rate among eligible patients in our primary care practice using the Web-based GDMS point-of-care clinical decision support tool. Early identification of patients with AAA allowed the patients to receive elective repair and avoid a potentially deadly aortic rupture.

In the context of a national shortage of primary care physicians, the immediate need seems to be to develop HIT systems that can provide clinical decision support. In addition, population-based technological systems may hold tremendous potential for allowing many preventive services and chronic disease management to move outside the busy primary care physician's office.

These systems can also identify tests that patients need prior to their visit and, if completed before the patient sees the provider, the provider can address them during the visit. Such systems and practice environment would benefit patients, providers and the entire health care system. However, barriers to HIT adoption, including cost and physician resistance to technology and information sharing, need to be addressed. As our country tackles health care reform, facilitating widespread adoption of technology such as clinical decision support through grants, incentives or mandates should be a priority.
